# Exploring the Effects of Antioxidants on αSynuclein-Induced Motor Deficits in *Drosophila* Larvae

**DOI:** 10.17912/micropub.biology.001897

**Published:** 2026-06-11

**Authors:** Sarah Perry, Aljwmared Zahraa, Evan Beard, Lorren Bonney, Logan Brown, Jacob Burkeen, Wendy Cardenas, Faith Clark, Gary Conner, Veronica Conrad, Melanie Freeman, Taylon Huycke, Jay Jernigan Cruz, Presley Jones, Sherrita Jones, Micah Kubr, Donnelle Kyles, KaMya McGuire, Kathleen Moore, Joel Phillips, Trinity Rains, Bradly Scharnhorst, Betty Smith, Hailey Stevens, Ryan Way

**Affiliations:** 1 Biology, Austin Peay State University, Clarksville, TN, United States; 2 Austin Peay State University, Clarksville, TN, US

## Abstract

Parkinson’s disease (PD) is marked by dopaminergic neuron loss, motor deficits, and oxidative stress. Misexpression of mutant human α-synuclein in *Drosophila* larvae induces PD-like motor impairments, making it a powerful and accessible model for studying neurodegeneration. In this Course-Based Undergraduate Research Experience (CURE), we used a novel Ring Maze assay to quantify larval locomotion following expression of A53T and E46K α-synuclein variants. Both induced robust crawling deficits. Notably, antioxidant treatment with vitamin C or açaí extract significantly improved motor performance. These findings demonstrate the value of the larval model for both PD research and hands-on student learning in neurobiology and therapeutic screening.

**Figure 1. Vitamin C and Acai treatment rescues motor deficits in a larval model of Parkinson’s disease:** A) Schematic depicting the Ring maze. A single wandering third instar larva is placed in the center of a 100mm 1% agarose plate placed atop a 60mm ring maze template. The amount of time, up to 60 seconds, it takes for the larvae to reach the edge of the ring is recorded. B) Failure rates for control (TH-Gal4/+) and α-synuclein larvae (TH-Gal4 > UAS-aSyn.E46K and TH-Gal4 > UAS-aSyn.A53T) with and without antioxidant treatments. Statistical comparison of % Failure was performed using a Chi-squared test for independence (N = 40-219). C) Mean time to edge for successful larvae in each genotype and treatment group. Error bars represent +/- SEM. Groups were compared using a Mann-Whitney U test (N = 29 – 141) D-F) Time to edge histograms for larvae of each genotype reared on various antioxidant treatments. Pairwise comparisons to the untreated groups were made using a Kolmogorov-Smirnov test (N = 40-219). (ns) p > 0.05, *p < 0.05, **p < 0.01, ***p < 0.001.

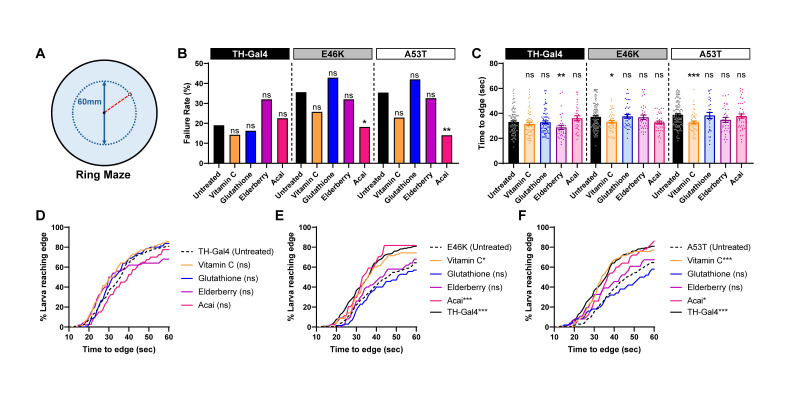



## Description

Parkinson’s disease (PD) is a progressive neurodegenerative disorder characterized by dopaminergic neuron (DAN) loss, motor dysfunction, and reduced lifespan (Aryal & Lee, 2019; Suzuki et al., 2022). A key hallmark of PD pathology is heightened oxidative stress and mitochondrial dysfunction, both of which contribute to neuronal degeneration (Aryal & Lee, 2019). Misexpression of mutant forms of human α-synuclein (αSyn), such as A53T and E46K, in *Drosophila melanogaster* larvae has been shown to recapitulate many PD-like phenotypes, including motor impairment, making the larval model an attractive system for studying disease mechanisms and testing therapeutic interventions (Blosser et al., 2020; Perry & More, 2025; Varga et al., 2014). In this study, conducted within a Course-Based Undergraduate Research Experience (CURE) framework, we explore the therapeutic potential of several antioxidant treatments using this larval PD model.

**Results** To develop a student-accessible yet biologically informative locomotion assay, we designed the Ring Maze—a simplified behavioral tool that captures αSyn-induced motor deficits in *Drosophila* larvae. Previous work from our lab showed that, in addition to reduced crawling speed, αSyn -expressing larvae exhibit impaired edge-seeking behavior (Perry & More, 2025). In standard 100mm agarose arenas, healthy larvae typically reach the edge of the arena within two minutes by crawling in a straight trajectory, whereas αSyn larvae often fail to navigate efficiently, exhibiting prolonged times to edge and frequent failure to reach the boundary.

Building on this, the Ring Maze consists of a 100mm 1% agarose arena placed over a template with a 60mm ring. A single wandering third instar larva is placed at the center, and the time taken to reach the edge of the 60mm ring is recorded (up to 60 seconds; Figure 1A ). More than 80% of control larvae successfully complete the maze within the time limit (failure rate: 19.01%), while larvae expressing human αSyn variants E46K and A53T show significantly increased failure rates of approximately 35% ( Figure 1B ; p = 0.000691 and p = 0.00087, Chi-squared test). Average time to edge for larvae that successfully completed the maze may also be compared. In this case, both E46K and A53T larvae displayed increased average time to edge compared to TH-Gal4 controls ( Figure 1C, p = 0.0042 and p < 0.0001, Mann-Whitney test). Time-to-edge data were also visualized using cumulative frequency plots to better illustrate locomotor differences across genotypes and treatments ( Figure 1D- F). For frequency distribution analysis, “failed” larvae were assigned a time to edge of 61 seconds. Using this approach, E46K and A53T larvae displayed poorer overall Ring Maze performance than TH-Gal4 larvae ( Figure 1D- F; p = 0.0001 and p < 0.0001, Kolmogorov Smirnov test). The different types of data collected provide an opportunity for students to use and interpret several types of statistical analysis.

To assess potential therapeutic interventions, we next tested whether dietary antioxidants could alleviate αSyn-related motor impairments. Based on their availability and prior associations with neuroprotection, we selected four treatments: vitamin C (250mg/L), glutathione (50mg/L), açaí extract (50mg/mL), and elderberry extract (50mg/mL). Of these, vitamin C and açaí supplementation significantly improved Ring Maze performance in both A53T and E46K larvae, enhancing navigational performance. In contrast, glutathione and elderberry showed no consistent benefit at the tested concentrations ( Figure 1C –E), suggesting that future studies might explore alternative dosing or combinatorial effects.

**Discussion**

These results highlight the utility of the *Drosophila* larval model for probing Parkinson’s disease (PD)-related motor deficits and screening potential therapeutic compounds. The Ring Maze assay is simple, cost-effective, and adaptable for undergraduate teaching, making it well suited for course-based research experiences that integrate experimental design and quantitative analysis with translational relevance.

Vitamin C’s neuroprotective effects in the context of PD have been previously established (Perry & More, 2025; Tran et al., 2018) and are largely attributed to its ability to reduce oxidative stress, a key contributor to α-synuclein toxicity. In contrast, the neuroprotective potential of acai is less well characterized. However, dietary acai has been previously shown in the fly system to convey protection against oxidative stress (Vrailas-Mortimer et al., 2012). Acai contains a range of antioxidant and anti-inflammatory phytochemicals, such as anthocyanins and flavonoids, and has been shown in other systems to reduce reactive oxygen species, modulate inflammatory signaling, and improve mitochondrial function. These processes are closely linked to PD pathology, suggesting plausible mechanisms for its effects (ALNasser & Mellor, 2022). While acai likely contains vitamin C, it’s primary antioxidants are anthocyanins (Matta et al., 2020; Yamaguchi et al., 2015) suggesting these might be interesting compounds to explore in future PD studies.

Together, our findings support the use of this model for rapid evaluation of dietary antioxidants and highlight the potential of less-characterized supplements like acai. Further work is needed to define their mechanisms of action and therapeutic potential in synucleopathy.

## Methods

Fly stocks and rearing: Flies were reared under standard conditions on cornmeal food (NutriFly, Bloomington formulation) at 25°C in a 12-hour light/dark cycle incubator. Larval density for crosses was controlled by pairing 6 females with 3-5 males and allowing the crosses to seed for 2-3 days before transferring the parents to a new tube. Wandering third instar larvae were typically observed on day 5 or 6 of culturing. Control genotypes were generated by outcrossing TH-Gal4 females to *w1118* males, and the resulting progeny were used for experiments. Fly stocks used in this study (obtained from the Bloomington Drosophila Stock Center, BDSC) were as follows: *w1118* (Perry lab stock), *TH-Gal4* (BDSC_8848), *UAS-E46K* (BDSC_80043), and *UAS-A53T* (BDSC_8148).

Antioxidant treatments: Antioxidant supplements were added to molten fly food to the following concentrations: Vitamin C (0.25mg/mL), Glutathione (0.05mg/mL), Acai extract (BulkSupplements, 50mg/mL), Elderberry extract (Nature’s Truth, 50mg/mL). Larvae were reared on treated food from egg to third instar.

Behavioral assays: The Ring maze is described in detail in the text and a user-friendly protocol is attached as supplement.

## Data Availability

Description: Ring Maze Protocol. Resource Type: InteractiveResource. DOI: https://doi.org/10.22002/63nnt-v1q07
